# Chlorido[1-(diphenyl­phosphan­yl)cobaltocenium]gold(I) hexa­fluoridophosphate

**DOI:** 10.1107/S1600536811018769

**Published:** 2011-05-25

**Authors:** Xiang-Hua Wu

**Affiliations:** a College of Chemistry and Chemical Engineering, Yunnan Normal University, Kunming 650092, People’s Republic of China

## Abstract

In the cobaltocenium group of the title compound, [AuCo(C_5_H_5_)(C_17_H_14_P)Cl]PF_6_, the substituted cyclo­penta­dienyl (Cps) and the unsubstituted cyclo­penta­dienyl (Cp) ring planes are almost parallel, making a dihedral angle of 3.1 (3)°. The C atoms in Cp and Cps are in an eclipsed conformation. The Au^I^ atom is coordinated by a P atom from the diphenyl­phosphanyl group and a Cl atom in an almost linear arrangement [P—Au—Cl = 178.15–(7)°]. Two hexa­fluorido­phosphate anions are each located on a twofold rotation axis. In the crystal, the complex cations and hexa­fluorido­phosphate anions are linked *via* inter­molecular C—H⋯F hydrogen bonds.

## Related literature

For a related structure, see: Chen *et al.* (2009 ▶).
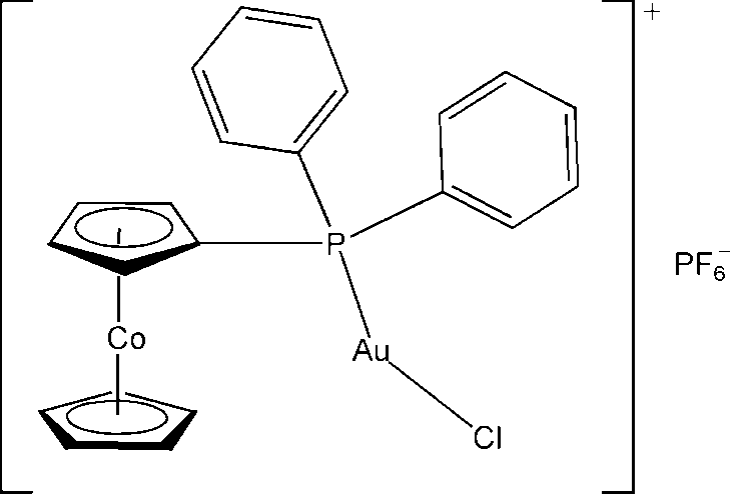


## Experimental

### 

#### Crystal data

[AuCo(C_5_H_5_)(C_17_H_14_P)Cl]PF_6_
*M* *_r_* = 750.66
Monoclinic, 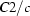

*a* = 11.5565 (14) Å
*b* = 14.8537 (14) Å
*c* = 27.983 (3) Å
β = 97.577 (2)°
*V* = 4761.6 (9) Å^3^
*Z* = 8
Mo *K*α radiation
μ = 7.15 mm^−1^
*T* = 292 K
0.20 × 0.20 × 0.20 mm

#### Data collection

Bruker APEX CCD diffractometer
24437 measured reflections
4680 independent reflections
4190 reflections with *I* > 2σ(*I*)
*R*_int_ = 0.183

#### Refinement

*R*[*F* ^2^ > 2σ(*F* ^2^)] = 0.048
*wR*(*F* ^2^) = 0.125
*S* = 1.06
4680 reflections
301 parameters
H-atom parameters constrained
Δρ_max_ = 3.36 e Å^−3^
Δρ_min_ = −1.59 e Å^−3^

### 

Data collection: *SMART* (Bruker, 2007 ▶); cell refinement: *SAINT* (Bruker, 2007 ▶); data reduction: *SAINT*; program(s) used to solve structure: *SHELXS97* (Sheldrick, 2008 ▶); program(s) used to refine structure: *SHELXL97* (Sheldrick, 2008 ▶); molecular graphics: *SHELXTL* (Sheldrick, 2008 ▶); software used to prepare material for publication: *SHELXTL*.

## Supplementary Material

Crystal structure: contains datablocks I, global. DOI: 10.1107/S1600536811018769/hy2423sup1.cif

Structure factors: contains datablocks I. DOI: 10.1107/S1600536811018769/hy2423Isup2.hkl

Additional supplementary materials:  crystallographic information; 3D view; checkCIF report

## Figures and Tables

**Table 1:** Hydrogen-bond geometry (Å, °)

*D*—H⋯*A*	*D*—H	H⋯*A*	*D*⋯*A*	*D*—H⋯*A*
C5—H5⋯F5^i^	0.98	2.56	3.249 (11)	128
C8—H8⋯F4^i^	0.98	2.41	3.090 (10)	126
C10—H10⋯F8^i^	0.98	2.53	3.185 (12)	125

Symmetry code: (i) 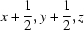.

**Fig. 1.**
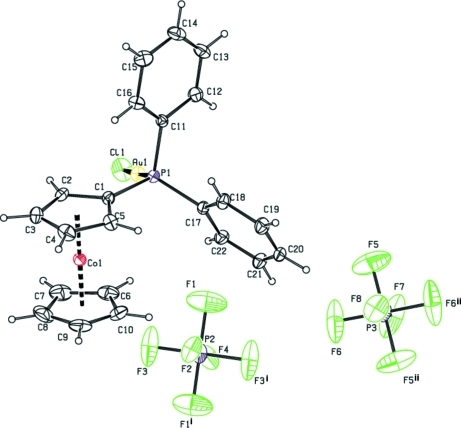
The molecular structure of the title compound, showing 30% probability displacement ellipsoids. [Symmetry codes: (i) 1-x, y, 1/2-z; (ii) -x, y, 1/2-z.]

**Fig. 2.**
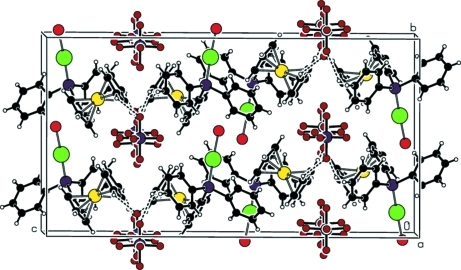
Crystal packing of the title compound, showing intermolecular C—H···F hydrogen bonds (dashed lines).

## supplementary crystallographic information

### Comment

The molecular structure of the title compound is shown in Fig. 1. In the cobaltocenium moiety (Chen *et al.*, 2009), two cyclopentadienyl rings are nearly parallel to each other with a dihedral angle at 3.1 (3)°. The C atoms of the substituted cyclopentadienyl (Cps) ring and the unsubstituted cyclopentadienyl (Cp) ring are in an eclipsed conformation. The Co1 atom is slightly nearer to the Cps plane, with the Co1—*Cgs* and Co1—*Cg* distances of 1.6275 (6) and 1.6323 (6) Å (*Cgs* and *Cg* are the centroids of the Cps and Cp rings). The *Cgs*—Co1—*Cg* angle is 177.06 (2)°. The P1, Au1 and Cl1 atoms are almost in a line, with an angle of 178.15 (7)°. The C1—P1—Au1 angle is 113.37 (18)°. The complex cations and hexafluorophosphate anions are linked by C—H···F hydrogen bonds (Table 1), as shown in Fig. 2.

### Experimental

To a solution of 1-(diphenylphosphanyl)cobaltocenium hexafluorophosphate (5.18 g, 0.01 mol) in dichloromethane (20 ml) was added gold chloride dimethylsulfane (0.62 g, 0.01 mol). The reaction mixture was stirred at room temperature for 30 min. After removing the solvent under reduced pressure, the residue was collected and dried in a vacuum desiccator (yield: 84%, 84 mg). Crystals suitable for X-ray data collection were obtained by slow evaporation from a dichloromethane and hexane solution at room temperature.

### Refinement

H atoms were positioned geometrically and refined as riding atoms, with C—H = 0.93 and 0.98 Å and with *U*_iso_(H) = 1.2*U*_eq_(C). The highest residual electron density was found at 0.83 Å from Au1 atom and the deepest hole at 1.33 Å from Au1 atom.

### Crystal data

**Table:**

[AuCo(C_5_H_5_)(C_17_H_14_P)Cl]PF_6_	*F*(000) = 2864
*M**_r_* = 750.66	*D*_x_ = 2.094 Mg m^−^^3^
Monoclinic, *C*2/*c*	Mo *K*α radiation, λ = 0.71073 Å
Hall symbol: -C 2yc	Cell parameters from 5908 reflections
*a* = 11.5565 (14) Å	θ = 2.2–27.5°
*b* = 14.8537 (14) Å	µ = 7.15 mm^−^^1^
*c* = 27.983 (3) Å	*T* = 292 K
β = 97.577 (2)°	Block, colorless
*V* = 4761.6 (9) Å^3^	0.20 × 0.20 × 0.20 mm
*Z* = 8	

### Data collection

**Table:**

Bruker APEX CCD diffractometer	4190 reflections with *I* > 2σ(*I*)
Radiation source: fine-focus sealed tube	*R*_int_ = 0.183
graphite	θ_max_ = 26.0°, θ_min_ = 2.3°
φ and ω scans	*h* = −14→14
24437 measured reflections	*k* = −18→18
4680 independent reflections	*l* = −34→34

### Refinement

**Table:**

Refinement on *F*^2^	Primary atom site location: structure-invariant direct methods
Least-squares matrix: full	Secondary atom site location: difference Fourier map
*R*[*F*^2^ > 2σ(*F*^2^)] = 0.048	Hydrogen site location: inferred from neighbouring sites
*wR*(*F*^2^) = 0.125	H-atom parameters constrained
*S* = 1.06	*w* = 1/[σ^2^(*F*_o_^2^) + (0.0706*P*)^2^] where *P* = (*F*_o_^2^ + 2*F*_c_^2^)/3
4680 reflections	(Δ/σ)_max_ = 0.001
301 parameters	Δρ_max_ = 3.36 e Å^−^^3^
0 restraints	Δρ_min_ = −1.59 e Å^−^^3^

### Fractional atomic coordinates and isotropic or equivalent isotropic displacement parameters (Å^2^)

**Table:**

	*x*	*y*	*z*	*U*_iso_*/*U*_eq_	
Au1	0.611737 (18)	0.111894 (15)	0.447818 (8)	0.04121 (13)	
Cl1	0.6800 (2)	−0.02937 (13)	0.46391 (9)	0.0810 (6)	
Co1	0.73961 (6)	0.31564 (5)	0.35937 (2)	0.0373 (2)	
C1	0.6515 (4)	0.3281 (4)	0.41722 (19)	0.0353 (11)	
C2	0.7751 (5)	0.3201 (4)	0.43184 (19)	0.0447 (13)	
H2	0.8134	0.2725	0.4524	0.054*	
C3	0.8318 (6)	0.3916 (5)	0.4118 (3)	0.0575 (19)	
H3	0.9161	0.4026	0.4157	0.069*	
C4	0.7441 (6)	0.4448 (5)	0.3837 (3)	0.0575 (17)	
H4	0.7581	0.4984	0.3649	0.069*	
C5	0.6348 (6)	0.4054 (4)	0.3868 (2)	0.0450 (13)	
H5	0.5597	0.4273	0.3707	0.054*	
C6	0.6927 (8)	0.1995 (6)	0.3237 (3)	0.070 (2)	
H6	0.6433	0.1519	0.3345	0.084*	
C7	0.8171 (8)	0.2057 (7)	0.3344 (3)	0.078 (2)	
H7	0.8690	0.1631	0.3535	0.094*	
C8	0.8503 (9)	0.2842 (7)	0.3112 (3)	0.088 (3)	
H8	0.9301	0.3066	0.3116	0.105*	
C9	0.7472 (9)	0.3263 (6)	0.2884 (2)	0.077 (2)	
H9	0.7434	0.3823	0.2697	0.092*	
C10	0.6548 (7)	0.2734 (7)	0.2960 (2)	0.070 (2)	
H10	0.5730	0.2868	0.2842	0.084*	
C11	0.5027 (4)	0.3014 (4)	0.48858 (17)	0.0323 (10)	
C12	0.4193 (6)	0.2539 (5)	0.5115 (2)	0.0525 (15)	
H12	0.3858	0.2012	0.4981	0.063*	
C13	0.3884 (6)	0.2873 (7)	0.5542 (2)	0.061 (2)	
H13	0.3328	0.2569	0.5693	0.074*	
C14	0.4375 (7)	0.3633 (6)	0.5745 (2)	0.0592 (17)	
H14	0.4159	0.3841	0.6034	0.071*	
C15	0.5198 (7)	0.4102 (5)	0.5526 (3)	0.0603 (17)	
H15	0.5537	0.4622	0.5667	0.072*	
C16	0.5513 (6)	0.3789 (4)	0.5092 (2)	0.0436 (14)	
H16	0.6056	0.4106	0.4941	0.052*	
C17	0.4166 (4)	0.2607 (4)	0.38938 (18)	0.0347 (11)	
C18	0.3477 (5)	0.3373 (5)	0.3875 (2)	0.0467 (14)	
H18	0.3665	0.3843	0.4090	0.056*	
C19	0.2490 (6)	0.3428 (6)	0.3524 (2)	0.0552 (17)	
H19	0.2033	0.3945	0.3500	0.066*	
C20	0.2205 (5)	0.2720 (6)	0.3221 (2)	0.0576 (19)	
H20	0.1534	0.2752	0.2998	0.069*	
C21	0.2892 (6)	0.1958 (6)	0.3239 (2)	0.0600 (18)	
H21	0.2698	0.1490	0.3022	0.072*	
C22	0.3869 (5)	0.1890 (5)	0.3580 (2)	0.0473 (14)	
H22	0.4325	0.1372	0.3599	0.057*	
F1	0.4729 (11)	0.0040 (5)	0.3031 (3)	0.183 (5)	
F2	0.5000	0.1112 (4)	0.2500	0.093 (3)	
F3	0.6304 (5)	0.0048 (5)	0.2686 (4)	0.168 (4)	
F4	0.5000	−0.1023 (4)	0.2500	0.094 (3)	
F5	0.0244 (12)	0.0474 (7)	0.3049 (3)	0.210 (5)	
F6	0.1298 (5)	0.0463 (7)	0.2511 (5)	0.226 (6)	
F7	0.0000	0.1482 (7)	0.2500	0.122 (3)	
F8	0.0000	−0.0631 (6)	0.2500	0.201 (7)	
P1	0.54402 (11)	0.25078 (9)	0.43452 (5)	0.0318 (3)	
P2	0.5000	0.00498 (18)	0.2500	0.0579 (6)	
P3	0.0000	0.0412 (2)	0.2500	0.0599 (7)	

### Atomic displacement parameters (Å^2^)

**Table:**

	*U*^11^	*U*^22^	*U*^33^	*U*^12^	*U*^13^	*U*^23^
Au1	0.04551 (17)	0.02754 (19)	0.05013 (19)	0.00192 (8)	0.00461 (11)	0.00108 (8)
Cl1	0.0891 (13)	0.0334 (10)	0.1220 (17)	0.0148 (9)	0.0195 (12)	0.0106 (10)
Co1	0.0351 (4)	0.0411 (5)	0.0363 (4)	0.0013 (3)	0.0067 (3)	0.0021 (3)
C1	0.036 (3)	0.030 (3)	0.039 (3)	−0.006 (2)	0.004 (2)	0.000 (2)
C2	0.042 (3)	0.056 (4)	0.034 (3)	−0.006 (3)	−0.005 (2)	−0.002 (2)
C3	0.045 (3)	0.067 (5)	0.060 (4)	−0.020 (3)	0.004 (3)	−0.006 (3)
C4	0.072 (4)	0.036 (4)	0.068 (4)	−0.010 (3)	0.024 (3)	0.001 (3)
C5	0.046 (3)	0.032 (3)	0.058 (3)	0.003 (2)	0.011 (3)	0.001 (3)
C6	0.105 (6)	0.054 (5)	0.054 (4)	−0.008 (4)	0.026 (4)	−0.020 (3)
C7	0.103 (7)	0.073 (6)	0.062 (4)	0.034 (5)	0.021 (4)	−0.011 (4)
C8	0.086 (6)	0.107 (8)	0.080 (5)	0.001 (5)	0.048 (5)	−0.026 (5)
C9	0.113 (7)	0.085 (6)	0.036 (3)	0.014 (5)	0.023 (4)	0.012 (4)
C10	0.077 (5)	0.091 (6)	0.039 (3)	−0.003 (4)	−0.008 (3)	−0.012 (4)
C11	0.031 (2)	0.033 (3)	0.034 (2)	0.000 (2)	0.0061 (19)	0.004 (2)
C12	0.053 (3)	0.059 (4)	0.046 (3)	−0.018 (3)	0.007 (3)	0.001 (3)
C13	0.061 (4)	0.078 (6)	0.050 (4)	−0.012 (3)	0.026 (3)	0.004 (3)
C14	0.068 (4)	0.066 (5)	0.047 (3)	0.014 (4)	0.023 (3)	−0.001 (3)
C15	0.079 (5)	0.044 (4)	0.058 (4)	0.004 (4)	0.009 (3)	−0.014 (3)
C16	0.056 (4)	0.035 (3)	0.042 (3)	0.001 (2)	0.014 (3)	0.001 (2)
C17	0.031 (2)	0.037 (3)	0.035 (2)	−0.003 (2)	0.0009 (19)	0.005 (2)
C18	0.042 (3)	0.045 (4)	0.051 (3)	0.001 (3)	−0.002 (2)	0.006 (3)
C19	0.042 (3)	0.065 (5)	0.057 (4)	0.012 (3)	−0.002 (3)	0.012 (3)
C20	0.035 (3)	0.098 (6)	0.038 (3)	−0.010 (3)	−0.003 (2)	0.010 (3)
C21	0.051 (4)	0.078 (5)	0.049 (3)	−0.021 (4)	−0.002 (3)	−0.009 (3)
C22	0.049 (3)	0.050 (4)	0.042 (3)	−0.009 (3)	0.003 (2)	−0.006 (3)
F1	0.356 (15)	0.085 (5)	0.129 (6)	0.025 (6)	0.108 (8)	−0.006 (4)
F2	0.094 (5)	0.045 (5)	0.127 (6)	0.000	−0.028 (5)	0.000
F3	0.070 (4)	0.088 (5)	0.328 (13)	0.000 (3)	−0.046 (6)	0.005 (6)
F4	0.102 (5)	0.039 (4)	0.152 (7)	0.000	0.059 (5)	0.000
F5	0.352 (15)	0.172 (10)	0.089 (5)	−0.019 (10)	−0.031 (7)	0.032 (5)
F6	0.061 (4)	0.173 (9)	0.439 (18)	−0.007 (4)	0.017 (7)	−0.151 (10)
F7	0.139 (8)	0.070 (6)	0.150 (8)	0.000	−0.009 (7)	0.000
F8	0.164 (9)	0.044 (5)	0.354 (19)	0.000	−0.116 (11)	0.000
P1	0.0328 (6)	0.0276 (7)	0.0340 (6)	−0.0005 (5)	0.0009 (5)	0.0011 (5)
P2	0.0468 (12)	0.0389 (14)	0.0854 (18)	0.000	−0.0007 (12)	0.000
P3	0.0431 (12)	0.0567 (17)	0.0755 (16)	0.000	−0.0085 (11)	0.000

### Geometric parameters (Å, °)

**Table:**

Au1—P1	2.2203 (14)	C11—P1	1.809 (5)
Au1—Cl1	2.2657 (18)	C12—C13	1.384 (9)
Co1—C9	2.005 (6)	C12—H12	0.9300
Co1—C10	2.010 (7)	C13—C14	1.354 (12)
Co1—C2	2.017 (5)	C13—H13	0.9300
Co1—C5	2.020 (6)	C14—C15	1.386 (11)
Co1—C7	2.030 (8)	C14—H14	0.9300
Co1—C6	2.030 (8)	C15—C16	1.392 (9)
Co1—C8	2.031 (7)	C15—H15	0.9300
Co1—C1	2.031 (5)	C16—H16	0.9300
Co1—C4	2.033 (7)	C17—C18	1.386 (8)
Co1—C3	2.037 (7)	C17—C22	1.394 (8)
C1—C5	1.426 (8)	C17—P1	1.815 (5)
C1—C2	1.437 (8)	C18—C19	1.404 (9)
C1—P1	1.805 (5)	C18—H18	0.9300
C2—C3	1.404 (9)	C19—C20	1.364 (11)
C2—H2	0.9800	C19—H19	0.9300
C3—C4	1.434 (11)	C20—C21	1.381 (11)
C3—H3	0.9800	C20—H20	0.9300
C4—C5	1.404 (9)	C21—C22	1.382 (9)
C4—H4	0.9800	C21—H21	0.9300
C5—H5	0.9800	C22—H22	0.9300
C6—C10	1.383 (12)	F1—P2	1.557 (7)
C6—C7	1.432 (13)	F2—P2	1.577 (7)
C6—H6	0.9800	F3—P2	1.528 (6)
C7—C8	1.413 (13)	F4—P2	1.594 (7)
C7—H7	0.9800	F5—P3	1.527 (8)
C8—C9	1.420 (13)	F6—P3	1.498 (6)
C8—H8	0.9800	F7—P3	1.589 (11)
C9—C10	1.364 (12)	F8—P3	1.549 (9)
C9—H9	0.9800	P2—F3^i^	1.528 (6)
C10—H10	0.9800	P2—F1^i^	1.557 (7)
C11—C16	1.374 (8)	P3—F6^ii^	1.498 (6)
C11—C12	1.414 (7)	P3—F5^ii^	1.526 (8)
			
P1—Au1—Cl1	178.15 (7)	C7—C8—Co1	69.6 (4)
C9—Co1—C10	39.7 (4)	C9—C8—Co1	68.4 (4)
C9—Co1—C2	164.5 (3)	C7—C8—H8	126.0
C10—Co1—C2	155.3 (3)	C9—C8—H8	126.0
C9—Co1—C5	115.6 (3)	Co1—C8—H8	126.0
C10—Co1—C5	107.1 (3)	C10—C9—C8	107.7 (8)
C2—Co1—C5	69.4 (3)	C10—C9—Co1	70.3 (4)
C9—Co1—C7	69.2 (4)	C8—C9—Co1	70.4 (4)
C10—Co1—C7	68.4 (4)	C10—C9—H9	126.1
C2—Co1—C7	109.4 (3)	C8—C9—H9	126.1
C5—Co1—C7	167.3 (4)	Co1—C9—H9	126.1
C9—Co1—C6	67.9 (4)	C9—C10—C6	110.4 (8)
C10—Co1—C6	40.0 (4)	C9—C10—Co1	69.9 (4)
C2—Co1—C6	121.9 (3)	C6—C10—Co1	70.8 (4)
C5—Co1—C6	127.8 (3)	C9—C10—H10	124.8
C7—Co1—C6	41.3 (4)	C6—C10—H10	124.8
C9—Co1—C8	41.2 (4)	Co1—C10—H10	124.8
C10—Co1—C8	67.6 (4)	C16—C11—C12	119.5 (5)
C2—Co1—C8	127.8 (4)	C16—C11—P1	124.1 (4)
C5—Co1—C8	150.0 (4)	C12—C11—P1	116.3 (4)
C7—Co1—C8	40.7 (4)	C13—C12—C11	118.8 (6)
C6—Co1—C8	68.2 (4)	C13—C12—H12	120.6
C9—Co1—C1	150.9 (3)	C11—C12—H12	120.6
C10—Co1—C1	119.6 (3)	C14—C13—C12	121.2 (6)
C2—Co1—C1	41.6 (2)	C14—C13—H13	119.4
C5—Co1—C1	41.2 (2)	C12—C13—H13	119.4
C7—Co1—C1	129.6 (3)	C13—C14—C15	120.6 (6)
C6—Co1—C1	109.9 (3)	C13—C14—H14	119.7
C8—Co1—C1	167.3 (4)	C15—C14—H14	119.7
C9—Co1—C4	104.8 (3)	C14—C15—C16	119.3 (7)
C10—Co1—C4	125.3 (4)	C14—C15—H15	120.3
C2—Co1—C4	68.8 (3)	C16—C15—H15	120.3
C5—Co1—C4	40.5 (3)	C11—C16—C15	120.5 (6)
C7—Co1—C4	151.8 (4)	C11—C16—H16	119.7
C6—Co1—C4	163.6 (4)	C15—C16—H16	119.7
C8—Co1—C4	116.7 (4)	C18—C17—C22	120.7 (5)
C1—Co1—C4	68.9 (2)	C18—C17—P1	120.2 (4)
C9—Co1—C3	125.6 (4)	C22—C17—P1	119.1 (4)
C10—Co1—C3	162.9 (4)	C17—C18—C19	119.0 (6)
C2—Co1—C3	40.5 (3)	C17—C18—H18	120.5
C5—Co1—C3	69.1 (3)	C19—C18—H18	120.5
C7—Co1—C3	118.7 (4)	C20—C19—C18	119.8 (7)
C6—Co1—C3	154.8 (4)	C20—C19—H19	120.1
C8—Co1—C3	106.9 (4)	C18—C19—H19	120.1
C1—Co1—C3	69.2 (2)	C19—C20—C21	121.3 (6)
C4—Co1—C3	41.3 (3)	C19—C20—H20	119.4
C5—C1—C2	106.7 (5)	C21—C20—H20	119.4
C5—C1—P1	128.9 (4)	C20—C21—C22	119.9 (7)
C2—C1—P1	124.3 (4)	C20—C21—H21	120.1
C5—C1—Co1	68.9 (3)	C22—C21—H21	120.1
C2—C1—Co1	68.7 (3)	C21—C22—C17	119.4 (6)
P1—C1—Co1	126.2 (3)	C21—C22—H22	120.3
C3—C2—C1	108.8 (6)	C17—C22—H22	120.3
C3—C2—Co1	70.5 (4)	C1—P1—C11	103.1 (2)
C1—C2—Co1	69.8 (3)	C1—P1—C17	106.6 (2)
C3—C2—H2	125.6	C11—P1—C17	105.6 (2)
C1—C2—H2	125.6	C1—P1—Au1	113.37 (18)
Co1—C2—H2	125.6	C11—P1—Au1	111.84 (17)
C2—C3—C4	107.6 (6)	C17—P1—Au1	115.32 (19)
C2—C3—Co1	69.0 (3)	F3^i^—P2—F3	179.8 (6)
C4—C3—Co1	69.2 (4)	F3^i^—P2—F1	90.7 (7)
C2—C3—H3	126.2	F3—P2—F1	89.3 (7)
C4—C3—H3	126.2	F3^i^—P2—F1^i^	89.3 (6)
Co1—C3—H3	126.2	F3—P2—F1^i^	90.7 (7)
C5—C4—C3	108.3 (6)	F1—P2—F1^i^	178.9 (6)
C5—C4—Co1	69.2 (4)	F3^i^—P2—F2	90.1 (3)
C3—C4—Co1	69.5 (4)	F3—P2—F2	90.1 (3)
C5—C4—H4	125.9	F1—P2—F2	90.5 (3)
C3—C4—H4	125.9	F1^i^—P2—F2	90.5 (3)
Co1—C4—H4	125.9	F3^i^—P2—F4	89.9 (3)
C4—C5—C1	108.7 (6)	F3—P2—F4	89.9 (3)
C4—C5—Co1	70.3 (4)	F1—P2—F4	89.5 (3)
C1—C5—Co1	69.8 (3)	F1^i^—P2—F4	89.5 (3)
C4—C5—H5	125.7	F2—P2—F4	180.000 (1)
C1—C5—H5	125.7	F6—P3—F6^ii^	174.2 (8)
Co1—C5—H5	125.7	F6—P3—F5^ii^	94.0 (7)
C10—C6—C7	107.5 (8)	F6^ii^—P3—F5^ii^	85.7 (7)
C10—C6—Co1	69.2 (5)	F6—P3—F5	85.7 (7)
C7—C6—Co1	69.3 (5)	F6^ii^—P3—F5	94.0 (7)
C10—C6—H6	126.2	F5^ii^—P3—F5	173.1 (9)
C7—C6—H6	126.2	F6—P3—F8	92.9 (4)
Co1—C6—H6	126.2	F6^ii^—P3—F8	92.9 (4)
C8—C7—C6	106.4 (8)	F5^ii^—P3—F8	93.4 (4)
C8—C7—Co1	69.7 (5)	F5—P3—F8	93.4 (4)
C6—C7—Co1	69.4 (4)	F6—P3—F7	87.1 (4)
C8—C7—H7	126.8	F6^ii^—P3—F7	87.1 (4)
C6—C7—H7	126.8	F5^ii^—P3—F7	86.6 (4)
Co1—C7—H7	126.8	F5—P3—F7	86.6 (4)
C7—C8—C9	107.9 (8)	F8—P3—F7	180.000 (2)
			
C9—Co1—C1—C5	−45.1 (8)	C8—Co1—C6—C7	38.5 (5)
C10—Co1—C1—C5	−82.2 (5)	C1—Co1—C6—C7	−128.0 (5)
C2—Co1—C1—C5	118.8 (5)	C4—Co1—C6—C7	149.4 (9)
C7—Co1—C1—C5	−167.7 (5)	C3—Co1—C6—C7	−44.8 (9)
C6—Co1—C1—C5	−125.2 (4)	C10—C6—C7—C8	−1.3 (8)
C8—Co1—C1—C5	155.8 (15)	Co1—C6—C7—C8	−60.2 (5)
C4—Co1—C1—C5	37.3 (4)	C10—C6—C7—Co1	58.9 (5)
C3—Co1—C1—C5	81.6 (4)	C9—Co1—C7—C8	37.6 (6)
C9—Co1—C1—C2	−163.9 (6)	C10—Co1—C7—C8	80.3 (6)
C10—Co1—C1—C2	159.0 (4)	C2—Co1—C7—C8	−126.0 (5)
C5—Co1—C1—C2	−118.8 (5)	C5—Co1—C7—C8	151.9 (13)
C7—Co1—C1—C2	73.6 (5)	C6—Co1—C7—C8	117.5 (7)
C6—Co1—C1—C2	116.0 (4)	C1—Co1—C7—C8	−168.4 (5)
C8—Co1—C1—C2	37.0 (16)	C4—Co1—C7—C8	−44.8 (9)
C4—Co1—C1—C2	−81.5 (4)	C3—Co1—C7—C8	−82.5 (6)
C3—Co1—C1—C2	−37.1 (4)	C9—Co1—C7—C6	−79.9 (5)
C9—Co1—C1—P1	78.4 (8)	C10—Co1—C7—C6	−37.1 (5)
C10—Co1—C1—P1	41.3 (5)	C2—Co1—C7—C6	116.6 (5)
C2—Co1—C1—P1	−117.7 (5)	C5—Co1—C7—C6	34.4 (16)
C5—Co1—C1—P1	123.6 (5)	C8—Co1—C7—C6	−117.5 (7)
C7—Co1—C1—P1	−44.1 (6)	C1—Co1—C7—C6	74.1 (6)
C6—Co1—C1—P1	−1.7 (5)	C4—Co1—C7—C6	−162.3 (6)
C8—Co1—C1—P1	−80.7 (16)	C3—Co1—C7—C6	160.0 (5)
C4—Co1—C1—P1	160.8 (4)	C6—C7—C8—C9	2.1 (9)
C3—Co1—C1—P1	−154.8 (4)	Co1—C7—C8—C9	−57.8 (5)
C5—C1—C2—C3	1.2 (7)	C6—C7—C8—Co1	59.9 (5)
P1—C1—C2—C3	180.0 (4)	C9—Co1—C8—C7	−120.0 (8)
Co1—C1—C2—C3	59.9 (4)	C10—Co1—C8—C7	−82.4 (6)
C5—C1—C2—Co1	−58.7 (4)	C2—Co1—C8—C7	75.1 (6)
P1—C1—C2—Co1	120.1 (4)	C5—Co1—C8—C7	−168.0 (6)
C9—Co1—C2—C3	30.1 (13)	C6—Co1—C8—C7	−39.1 (5)
C10—Co1—C2—C3	−167.9 (7)	C1—Co1—C8—C7	44.7 (18)
C5—Co1—C2—C3	−81.6 (5)	C4—Co1—C8—C7	158.1 (5)
C7—Co1—C2—C3	111.9 (5)	C3—Co1—C8—C7	114.7 (6)
C6—Co1—C2—C3	155.9 (5)	C10—Co1—C8—C9	37.6 (6)
C8—Co1—C2—C3	70.0 (6)	C2—Co1—C8—C9	−164.9 (5)
C1—Co1—C2—C3	−119.7 (6)	C5—Co1—C8—C9	−48.0 (10)
C4—Co1—C2—C3	−38.0 (4)	C7—Co1—C8—C9	120.0 (8)
C9—Co1—C2—C1	149.8 (11)	C6—Co1—C8—C9	80.9 (6)
C10—Co1—C2—C1	−48.2 (9)	C1—Co1—C8—C9	164.7 (13)
C5—Co1—C2—C1	38.1 (3)	C4—Co1—C8—C9	−81.9 (6)
C7—Co1—C2—C1	−128.4 (4)	C3—Co1—C8—C9	−125.3 (6)
C6—Co1—C2—C1	−84.4 (5)	C7—C8—C9—C10	−2.2 (9)
C8—Co1—C2—C1	−170.3 (5)	Co1—C8—C9—C10	−60.7 (5)
C4—Co1—C2—C1	81.6 (4)	C7—C8—C9—Co1	58.5 (5)
C3—Co1—C2—C1	119.7 (6)	C2—Co1—C9—C10	168.4 (10)
C1—C2—C3—C4	−0.8 (7)	C5—Co1—C9—C10	−86.3 (6)
Co1—C2—C3—C4	58.7 (5)	C7—Co1—C9—C10	80.9 (6)
C1—C2—C3—Co1	−59.4 (4)	C6—Co1—C9—C10	36.4 (5)
C9—Co1—C3—C2	−170.5 (4)	C8—Co1—C9—C10	118.0 (8)
C10—Co1—C3—C2	162.6 (10)	C1—Co1—C9—C10	−55.1 (9)
C5—Co1—C3—C2	82.4 (4)	C4—Co1—C9—C10	−128.1 (5)
C7—Co1—C3—C2	−86.6 (5)	C3—Co1—C9—C10	−168.0 (5)
C6—Co1—C3—C2	−54.5 (8)	C10—Co1—C9—C8	−118.0 (8)
C8—Co1—C3—C2	−129.1 (5)	C2—Co1—C9—C8	50.3 (14)
C1—Co1—C3—C2	38.1 (4)	C5—Co1—C9—C8	155.7 (6)
C4—Co1—C3—C2	119.4 (6)	C7—Co1—C9—C8	−37.2 (6)
C9—Co1—C3—C4	70.1 (5)	C6—Co1—C9—C8	−81.7 (6)
C10—Co1—C3—C4	43.1 (12)	C1—Co1—C9—C8	−173.1 (6)
C2—Co1—C3—C4	−119.4 (6)	C4—Co1—C9—C8	113.8 (6)
C5—Co1—C3—C4	−37.1 (4)	C3—Co1—C9—C8	73.9 (7)
C7—Co1—C3—C4	154.0 (4)	C8—C9—C10—C6	1.4 (9)
C6—Co1—C3—C4	−174.0 (6)	Co1—C9—C10—C6	−59.4 (5)
C8—Co1—C3—C4	111.5 (5)	C8—C9—C10—Co1	60.8 (5)
C1—Co1—C3—C4	−81.3 (4)	C7—C6—C10—C9	−0.1 (9)
C2—C3—C4—C5	0.0 (8)	Co1—C6—C10—C9	58.9 (6)
Co1—C3—C4—C5	58.5 (5)	C7—C6—C10—Co1	−58.9 (5)
C2—C3—C4—Co1	−58.5 (5)	C2—Co1—C10—C9	−172.6 (6)
C9—Co1—C4—C5	112.2 (5)	C5—Co1—C10—C9	109.6 (5)
C10—Co1—C4—C5	74.2 (5)	C7—Co1—C10—C9	−83.0 (6)
C2—Co1—C4—C5	−82.6 (4)	C6—Co1—C10—C9	−121.3 (7)
C7—Co1—C4—C5	−174.4 (6)	C8—Co1—C10—C9	−38.9 (6)
C6—Co1—C4—C5	50.9 (12)	C1—Co1—C10—C9	152.7 (5)
C8—Co1—C4—C5	154.6 (5)	C4—Co1—C10—C9	68.7 (6)
C1—Co1—C4—C5	−37.9 (4)	C3—Co1—C10—C9	35.2 (13)
C3—Co1—C4—C5	−120.0 (6)	C9—Co1—C10—C6	121.3 (7)
C9—Co1—C4—C3	−127.8 (5)	C2—Co1—C10—C6	−51.3 (9)
C10—Co1—C4—C3	−165.8 (4)	C5—Co1—C10—C6	−129.1 (5)
C2—Co1—C4—C3	37.4 (4)	C7—Co1—C10—C6	38.3 (5)
C5—Co1—C4—C3	120.0 (6)	C8—Co1—C10—C6	82.4 (6)
C7—Co1—C4—C3	−54.4 (8)	C1—Co1—C10—C6	−86.0 (5)
C6—Co1—C4—C3	170.9 (9)	C4—Co1—C10—C6	−170.0 (4)
C8—Co1—C4—C3	−85.4 (5)	C3—Co1—C10—C6	156.5 (10)
C1—Co1—C4—C3	82.1 (4)	C16—C11—C12—C13	0.3 (9)
C3—C4—C5—C1	0.8 (8)	P1—C11—C12—C13	177.0 (6)
Co1—C4—C5—C1	59.5 (4)	C11—C12—C13—C14	−0.9 (11)
C3—C4—C5—Co1	−58.7 (5)	C12—C13—C14—C15	0.5 (13)
C2—C1—C5—C4	−1.2 (7)	C13—C14—C15—C16	0.4 (12)
P1—C1—C5—C4	−179.9 (5)	C12—C11—C16—C15	0.6 (9)
Co1—C1—C5—C4	−59.7 (5)	P1—C11—C16—C15	−175.9 (5)
C2—C1—C5—Co1	58.5 (4)	C14—C15—C16—C11	−1.0 (11)
P1—C1—C5—Co1	−120.2 (5)	C22—C17—C18—C19	−1.7 (9)
C9—Co1—C5—C4	−82.8 (5)	P1—C17—C18—C19	−179.4 (5)
C10—Co1—C5—C4	−124.7 (5)	C17—C18—C19—C20	2.1 (10)
C2—Co1—C5—C4	81.2 (4)	C18—C19—C20—C21	−2.4 (10)
C7—Co1—C5—C4	168.0 (13)	C19—C20—C21—C22	2.1 (10)
C6—Co1—C5—C4	−163.9 (5)	C20—C21—C22—C17	−1.7 (9)
C8—Co1—C5—C4	−50.0 (9)	C18—C17—C22—C21	1.5 (8)
C1—Co1—C5—C4	119.6 (6)	P1—C17—C22—C21	179.2 (5)
C3—Co1—C5—C4	37.7 (4)	C5—C1—P1—C11	−89.4 (5)
C9—Co1—C5—C1	157.5 (4)	C2—C1—P1—C11	92.2 (5)
C10—Co1—C5—C1	115.7 (4)	Co1—C1—P1—C11	179.5 (3)
C2—Co1—C5—C1	−38.4 (3)	C5—C1—P1—C17	21.5 (6)
C7—Co1—C5—C1	48.3 (15)	C2—C1—P1—C17	−156.9 (5)
C6—Co1—C5—C1	76.5 (5)	Co1—C1—P1—C17	−69.6 (4)
C8—Co1—C5—C1	−169.6 (7)	C5—C1—P1—Au1	149.5 (5)
C4—Co1—C5—C1	−119.6 (6)	C2—C1—P1—Au1	−29.0 (5)
C3—Co1—C5—C1	−81.9 (4)	Co1—C1—P1—Au1	58.4 (4)
C9—Co1—C6—C10	−36.1 (5)	C16—C11—P1—C1	−2.2 (6)
C2—Co1—C6—C10	157.4 (5)	C12—C11—P1—C1	−178.8 (4)
C5—Co1—C6—C10	69.9 (6)	C16—C11—P1—C17	−113.9 (5)
C7—Co1—C6—C10	−119.2 (7)	C12—C11—P1—C17	69.5 (5)
C8—Co1—C6—C10	−80.6 (6)	C16—C11—P1—Au1	120.0 (5)
C1—Co1—C6—C10	112.8 (5)	C12—C11—P1—Au1	−56.6 (5)
C4—Co1—C6—C10	30.2 (12)	C18—C17—P1—C1	−71.4 (5)
C3—Co1—C6—C10	−164.0 (6)	C22—C17—P1—C1	110.9 (4)
C9—Co1—C6—C7	83.1 (5)	C18—C17—P1—C11	37.8 (5)
C10—Co1—C6—C7	119.2 (7)	C22—C17—P1—C11	−139.9 (4)
C2—Co1—C6—C7	−83.4 (5)	C18—C17—P1—Au1	161.8 (4)
C5—Co1—C6—C7	−171.0 (5)	C22—C17—P1—Au1	−15.9 (5)

Symmetry codes: (i) −*x*+1, *y*, −*z*+1/2; (ii) −*x*, *y*, −*z*+1/2.

### Hydrogen-bond geometry (Å, °)

**Table:**

*D*—H···*A*	*D*—H	H···*A*	*D*···*A*	*D*—H···*A*
C5—H5···F5^iii^	0.98	2.56	3.249 (11)	128
C8—H8···F4^iii^	0.98	2.41	3.090 (10)	126
C10—H10···F8^iii^	0.98	2.53	3.185 (12)	125

Symmetry codes: (iii) *x*+1/2, *y*+1/2, *z*.
